# Fibrinogen-to-Albumin Ratio and Blood Urea Nitrogen-to-Albumin Ratio in COVID-19 Patients: A Systematic Review and Meta-Analysis

**DOI:** 10.3390/tropicalmed7080150

**Published:** 2022-07-27

**Authors:** Juan R. Ulloque-Badaracco, Esteban A. Alarcon-Braga, Enrique A. Hernandez-Bustamante, Ali Al-kassab-Córdova, Melany D. Mosquera-Rojas, Ricardo R. Ulloque-Badaracco, Miguel A. Huayta-Cortez, Sherelym H. Maita-Arauco, Percy Herrera-Añazco, Vicente A. Benites-Zapata

**Affiliations:** 1 Escuela de Medicina, Universidad Peruana de Ciencias Aplicadas, Lima 15023, Peru; ramiroullobada@gmail.com (J.R.U.-B.); aealarco@gmail.com (E.A.A.-B.); aliac1998@gmail.com (A.A.-k.-C.); mosqueramelany5@gmail.com (M.D.M.-R.); miguel.huayta@hotmail.com (M.A.H.-C.); harumimaita3@gmail.com (S.H.M.-A.); 2 Sociedad Científica de Estudiantes de Medicina de la Universidad Peruana de Ciencias Aplicadas, Lima 15023, Peru; 3 Sociedad Científica de Estudiantes de Medicina de la Universidad Nacional de Trujillo, Trujillo 13011, Peru; eahernandezbustamante@gmail.com; 4 Grupo Peruano de Investigación Epidemiológica, Unidad Para la Generación y Síntesis de Evidencias en Salud, Universidad San Ignacio de Loyola, Lima 15012, Peru; 5 Facultad de Ciencias de la Salud, Universidad Científica del Sur, Lima 15067, Peru; ricardoulloque483@gmail.com; 6 Escuela de Enfermería, Universidad Privada San Juan Bautista, Lima 15067, Peru; silamud@gmail.com; 7 Instituto de Evaluación de Tecnologías en Salud e Investigación—IETSI, EsSalud, Lima 14072, Peru; 8 Unidad de Investigación Para la Generación y Síntesis de Evidencias en Salud, Universidad San Ignacio de Loyola, Lima 15012, Peru

**Keywords:** COVID-19, fibrinogen, albumin, urea, blood urea nitrogen

## Abstract

Fibrinogen-to-albumin ratio (FAR) and blood urea nitrogen-to-albumin ratio (BAR) are inflammatory biomarkers that have been associated with clinical outcomes of multiple diseases. The objective of this study is to evaluate the association of these biomarkers with the severity and mortality of COVID-19 patients. A systematic search was performed in five databases. Observational studies that reported the association between FAR and BAR values with the severity and mortality of COVID-19 patients were included. Random-effects models were used for meta-analyses, and effects were expressed as Odds Ratio (OR) and their 95% confidence intervals (CI). Publication bias was assessed using the Begg test, while the quality assessment was assessed using the Newcastle Ottawa Scale. A total of 21 studies (n = 7949) were included. High FAR values were associated with a higher risk of severity (OR: 2.41; 95% CI 1.41–4.12; *p* < 0.001) and mortality (OR: 2.05; 95% CI 1.66–2.54; *p* < 0.001). High BAR values were associated with higher risk of mortality (OR: 4.63; 95% CI 2.11–10.15; *p* < 0.001). However, no statistically significant association was found between BAR values and the risk of severity (OR: 1.16; 95% CI 0.83–1.63; *p* = 0.38). High FAR and BAR values were associated with poor clinical outcomes.

## 1. Introduction

The COVID-19 pandemic has put health systems in check so that to date there are more than 500 million confirmed cases and 6 million deaths [1]. The high demand for specialized health care services together with the low supply of these, especially in low- and middle-income countries, has worsened the global panorama [2]. The consequences of the pandemic are not limited to the health sector but have also severely affected economies, education, and other fields [3,4]. In this sense, it is important to have tools, such as biomarkers, which allow the medical task force to quickly predict severity among critically ill patients.

Several biomarkers have been proven to predict severity and mortality among COVID-19 patients. For instance, neutrophil-to-lymphocyte ratio, procalcitonin, D-dimer, interleukin-6, ferritin, and apolipoproteins, among others [5,6,7,8,9,10]. Nevertheless, some of these might be imprecise, expensive, and limited in certain developing countries [11]. Both fibrinogen-to-albumin ratio (FAR) and blood urea nitrogen-to-albumin ratio (BAR) are potential alternatives to predict severity in COVID-19 patients. They are widely accessible, simple, and economical. 

FAR is a quotient extensively used to predict disease progression. Fibrinogen is a positive acute-phase reactant involved in coagulation and thrombosis and has been associated with excessive inflammation in COVID-19 patients [12]. Meanwhile, albumin is a negative acute-phase reactant [13]. The elevation of the fibrinogen along with the decrease of albumin is common in inflammatory diseases. It has been demonstrated to be effective in predicting severity and mortality in certain medical conditions such as cancer, cardiovascular and cerebrovascular disorders, among others [14,15,16,17].

BAR is a predictive marker employed in several medical conditions such as pneumonia, cardiovascular and gastrointestinal diseases [18,19,20,21]. The kidney involvement, which led to elevated blood urea nitrogen levels, is highly prevalent among COVID-19 patients due to an uncontrolled systemic immune response [22]. As previously mentioned, the elevation of blood urea nitrogen along with the decrease of albumin is a laboratory condition produced by high inflammatory states such as COVID-19.

Although vaccines have been shown to significantly reduce the mortality of patients with COVID-19 [23], the growing threat of new genomic variants of concern demands prognostic tools for those patients susceptible to complications [24]. Recently, several articles have been published which assess the prognostic role of the above-described biomarkers on the outcome of COVID-19 patients. We, therefore, aimed to conduct a systematic review and meta-analysis of observational studies addressing the prognostic value of FAR and BAR among patients with COVID-19.

## 2. Methods

We followed the Preferred Reporting Items for Systematic Reviews and Meta-analysis (PRISMA) statement [25] for drafting this systematic review (See PRISMA checklist in Supplementary Table S1) and submitted a summarized version of the protocol to the International Prospective Register of Systematic Review (PROSPERO) [CRD42022326416].

### 2.1. Search Strategy and Databases

We follow the Peer Review of Electronic Search Strategies (PRESS) Guidelines [26] for building the search strategy, which is attached as supplemental material (see Search Strategy in Supplementary Table S2). On 7 June 2022 we run the systematic search in five databases (PubMed, Web of Science, Ovid Medline, Embase, and Scopus) with no language restriction. A manual search was also carried out on preprint platforms (MedRxiv, Research Square and BioRxiv).

### 2.2. Eligibility Criteria

We searched for studies assessing the association between FAR or BAR, and the severity or mortality of COVID-19. Inclusion criteria were (a) studies with case-control or cohort designs that (b) enrolled adult patients (≥18 years) and (c) who had been diagnosed to have COVID-19. Our primary outcomes were severity and mortality.

### 2.3. Study Selection Process and Data Extraction

We exported all retrieved references from databases to Rayyan QCRI (Rayyan Systems Inc. ©, Cambridge, MA, USA) [27]. After removing duplicates, four authors (J.R.U.-B., E.A.A.-B., E.A.H.-B. and R.R.U.-B.) performed independently the screening by title and abstracts. Likewise, these authors independently reviewed the remaining references in full-text. References meeting all eligibility criteria at full-text screening were finally included. We resolved any conflict on decisions at any stage of the study selection process by consensus. Afterwards, two authors (S.H.M.-A. and M.A.H.-C.) independently extracted the information required for data synthesis from the included studies in a Microsoft Excel © template. Any conflict regarding the extracted information was resolved through consensus. We extracted the following data: first author, publication date, study title, study design, study location, number of participants, age, sex, BAR, FAR, outcomes (mortality or severity) and association measures (crude or adjusted with their 95% confidence intervals).

### 2.4. Quality Assessment

Two authors (A.A.-k.-C. and M.D.M.-R.) independently assessed the risk of bias of all included studies with the Newcastle-Ottawa Scale (NOS) [28]. The risk of bias was categorized as low (≥6 stars) and high (≤5 stars).

### 2.5. Assessment of Publication Bias

We evaluated publication bias with Begg test [29]. No publication bias was considered if the *p*-value was greater than 0.1.

### 2.6. Statistical Analyses

We transformed continuous data presented as medians and their respective interquartile ranges (IQR) into means and standard deviations (SD), respectively, according to Hozo method [30]. The only measure of association used was the Odds Ratio (OR) and its 95% confidence intervals (CI). In this sense, the standardized mean differences were transformed into ln [OR] using the Chinn method [31], and the Hazard Ratio (HR) was also converted into OR [32]. Review Manager 5.4 was used to perform a random-effects model meta-analysis. This model was selected because we anticipated heterogeneity between studies. Statistical heterogeneity was assessed using I^2^ statistics and the Cochran Q test. We categorized the I^2^ test as severe (≥60%) and non-severe (<60%). We performed a subgroup analysis by country. In addition, a sensitivity analysis using only studies with a low risk of bias was performed.

## 3. Results

### 3.1. Search Results

**Figure 1:**
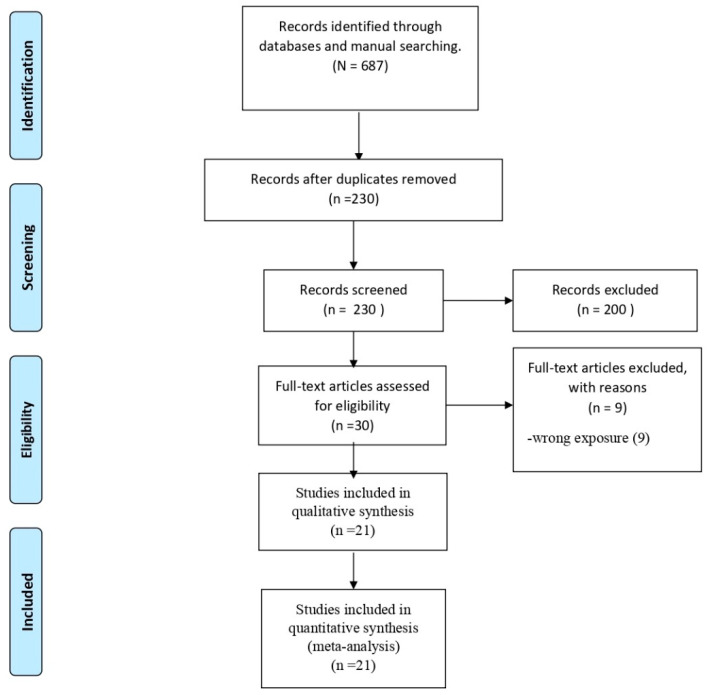
PRISMA Flow Diagram.

Databases search found 687 studies; from those, 457 were eliminated because of duplication. In the screening according to titles and abstracts of the remaining 230 articles, 200 studies were excluded. Then, a total of 30 studies were eligible for full-text screening where 9 articles were excluded. In the end, 21 studies were included for the systemic review and metanalyses [33,34,35,36,37,38,39,40,41,42,43,44,45,46,47,48,49,50,51,52,53]. The flowchart of the selection process is shown in Figure 1.

### 3.2. Study Characteristics

A total of 21 cohort studies were included, thirteen of which were from Turkey, four in China, one from Nigeria, one from India, one from Iran and one from Croatia. Of those, eight studies only evaluated severity, nine studies only evaluated mortality and four studies evaluated both outcomes. 

Of the 7949 patients included in the study, 3822 (48.08%) were male, whose ages ranged from 22 to 88 years. The quality assessment showed that ten articles had a low risk of bias while the other eleven articles had a high risk of bias (Table S3). The characteristics of each study are summarized in Table 1 and Table 2.

### 3.3. Association between FAR Values and Severity of COVID-19 Patients

**Figure 2:**
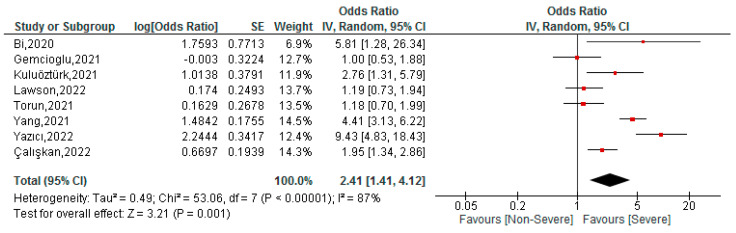
Association between FAR and severity of COVID-19 patients [33,34,36,39,40,41,50,52].

This association was assessed in eight studies with a total of 2897 patients. The cut-off ranged from 0.088 to 0.15, while the area under the curve (AUC) ranged between 0.629 to 0.838. In the meta-analysis we found that COVID-19 patients with high FAR values had a higher risk of severe disease (OR: 2.41; 95% CI 1.41–4.12; *p* < 0.001) (Figure 2). Given the high heterogeneity of the studies (I^2^ = 87%), we performed a subgroup analysis by country (Figure S1), where the association remained in the Turkish studies (OR: 2.22; 95% CI 1.11–4.45; *p* < 0.001; I^2^ = 87%) and in the Chinese studies (OR: 4.47; 95% CI 3.2–6.25; *p* < 0.001; I^2^ = 0%). In sensitivity analysis, in which we just included studies with a low risk of bias (Figure S2), less heterogeneity and an increase in the magnitude of the association was found (OR: 4.12; 95% CI 3.03–5.59; *p* < 0.001; I^2^ = 0%).

### 3.4. Association between FAR Values and Mortality of COVID-19 Patients

**Figure 3:**
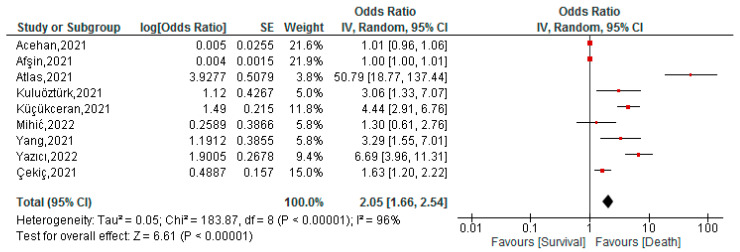
Association between FAR and mortality of COVID-19 patients [34,35,37,38,40,41,42,43,52].

This association was assessed in nine studies with a total of 3693 patients. The cut-off ranged from 0.111 to 0.15, while the AUC ranged from 0.654 to 0.989. In the meta-analysis we found that COVID-19 patients with high FAR values had a higher risk of mortality (OR: 2.05; 95% CI 1.66–2.54; *p* < 0.001) (Figure 3). Given the high heterogeneity (I^2^ = 96%), we performed a subgroup analysis by country (Figure S3), and no decrease in heterogeneity in the Turkish subgroup was found (OR: 2.03; 95% CI 1.62–2.54; *p* < 0.001; I^2^ = 97%). In sensitivity analysis, in which we just included studies with a low risk of bias (Figure S4), less heterogeneity was found and the association remained (OR: 1.99; 95% CI 1.33–2.98; *p* < 0.001; I^2^ = 41%).

### 3.5. Association between BAR Values and Severity of COVID-19 Patients

**Figure 4:**
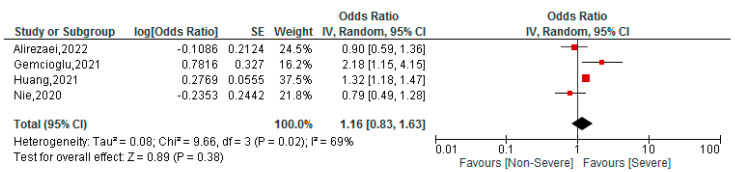
Association between BAR and severity of COVID-19 patients [33,44,45,49].

This association was assessed in four studies with a total of 2201 patients. The cut-off ranged from 3.788 to 4.78, while the AUC ranged from 0.475 to 0.821. In the meta-analysis, no statistically significant association was found between BAR values and the risk of severe disease (OR: 1.16; 95% CI 0.83–1.63; *p* = 0.38) (Figure 4).

### 3.6. Association between BAR Values and Mortality of COVID-19 Patients

**Figure 5:**
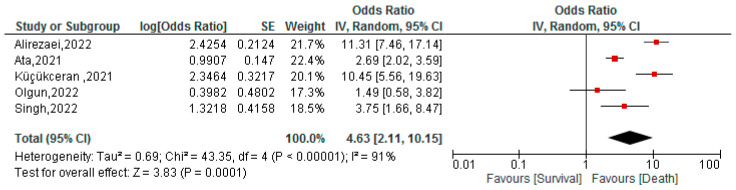
Association between BAR and mortality of COVID-19 patients [46,47,48,49,51].

This association was assessed in 1756 patients in five studies. The cut-off ranged from 3.4 to 6.23, while the AUC ranged from 0.695 to 0.823. In the meta-analysis, we found that COVID-19 patients with high FAR values were associated with a higher risk of mortality (OR: 4.63; 95% CI 2.11–10.15; *p* < 0.001) (Figure 5).

### 3.7. Publication Bias

In the Begg test, no publication bias was found between the association of FAR with severity (*p* = 0.7105) and mortality (*p* = 0.1753). Likewise, there was not publication bias between BAR and mortality (*p* = 1.5376).

## 4. Discussion

The main results of our study show that high FAR values were associated with a higher risk of disease severity and mortality, and that high BAR values were associated with a higher risk of mortality in patients with COVID-19.

Both FAR and BAR are indirect markers of inflammation that are good predictors in patients with conditions where inflammation is an important factor. In patients with coronary disease, Çetin et al. showed the predictive value of FAR on the development of major cardiovascular events in patients treated with percutaneous coronary intervention for acute coronary syndrome [14]. Liu et al. found that FAR was a good predictor of long-term outcomes in patients with ST-segment elevation myocardial infarction and multivessel disease [15]. In cancer patients, FAR was also shown to be a good predictor of all-cause mortality [54]. Additionally, FAR is a predictor of diabetic kidney disease with better performance than fibrinogen and albumin alone [55]. It also reflects the activity of ANCA-associated vasculitis [56], or the acute-phase response after total knee arthroplasty [57]. Similarly, BAR was shown to be a predictor of long-term mortality in patients with acute myocardial infarction in the intensive care unit [58], as well as hospital mortality in older adults [59] or patients with acute pulmonary thromboembolism [60].

Concerning infectious diseases, both biomarkers have also been shown to have predictive properties. BAR independently predicts 30-day mortality and severity in patients with Escherichia coli bacteremia [61]. In the same way, FAR was shown to be a predictor of events in patients with pneumonia. Indeed, a systematic review showed that BAR is a prognostic factor for various types of pneumonia [18] and another study showed that FAR predicts severity among patients with community-acquired pneumonia [62]. In this sense, our results reaffirm the prognostic value of these biomarkers in patients with lung infections (in this case due to COVID-19), where inflammation plays a role as a marker of severity and poor prognosis [63]. Certainly, among patients with severe COVID-19, elevated values of procalcitonin, C-reactive protein, D-dimer, and lactic dehydrogenase have been found [64]. Likewise, in these patients, there was a marked decrease in the values of lymphocytes, monocytes, eosinophils and platelets [64,65,66,67]. Other novel biomarkers of inflammation constructed with the values of these elements have also been shown to be equally useful, such as the albumin to globulin ratio, C-reactive protein/albumin ratio, lymphocyte/monocyte ratio, among others [68,69,70].

BAR and FAR are constructed based on values of fibrinogen, albumin and urea, which are altered in patients with COVID-19. Fibrinogen is one of the acute phase proteins that is synthesized by the liver in response to stimulation by IL-1 and IL-6. Fibrinogen participates in the formation of fibrin as the last step in the coagulation process, and is used as one of the scoring parameters in the diagnosis of disseminated intravascular coagulation [71,72]. Several studies indicated that the level of fibrinogen and its degradation products is not only higher in patients with COVID-19 compared to healthy patients, but also higher in critically ill patients compared to mild or moderate patients [12,73,74,75]. In the case of albumin, it is more susceptible to non-enzymatic glycation reactions and regulates the expression of ACE2, which is the target receptor of COVID-19 [70]. Hypoalbuminemia has been linked to COVID-19 and appears to predict outcomes regardless of age and morbidity [76]. A systematic review found that serum albumin concentration was significantly lower in patients with severe disease and was significantly associated with disease severity and adverse outcomes in these patients [77].

It was noteworthy that high values of BAR were not associated with the severity of the disease. However, this is likely due to the conditions under which urea was tested. A high BAR value may be associated with a high urea value, which may be a consequence of acute kidney injury, a frequent complication in patients with COVID-19 [78]. In patients with acute kidney injury, it is possible to find fluid overload from the initial stages [79], which means that in severe cases that do not respond to fluid restriction and the use of diuretics, renal replacement therapy is indicated [80,81]. For this reason, the association between high urea values and higher mortality in patients with acute kidney injury [82,83] could explain our findings. However, the presence of high BAR values in patients without the severe disease can be explained by the units of measurement used or the dilution of urea values caused by fluid overload, similarly to what happens with the serum creatinine [84,85]. Consequently, it is necessary to point out the importance of considering the level of water overload for a correct estimation of BAR values [86].

To the best of our knowledge, this is the first systematic review and meta-analysis evaluating the association of these biomarkers in COVID-19 patients. Besides, NOS was employed to assess the risk of bias among the included articles and sensibility analysis was performed considering the bias of the studies, which gives robustness to our results. Even though infections have decreased in many countries [1], the emergence of new variants [87] and new waves of infections in Asian countries [88], suggest that the world’s health systems will continue treating COVID-19 patients in their hospitals. Therefore, our findings let us suggest potential biomarkers of low cost that will allow health personnel to prioritize or individualize management strategies in patients with COVID-19.

Although the usefulness of various markers [5,89], including composite biomarkers [10,63,64,65], in the prognosis of patients with COVID-19 has been evaluated, their applicability in clinical practice remains conceptual due to flaws in the design of their studies. Most studies are retrospective with different cut-off points, measurement time points, chosen endpoints, and although many studies adjusted their analysis for various factors, unmeasured confounders cannot be excluded, limiting the utility of a biomarker in predicting the prognosis of these patients [90]. Regarding meta-analyses, the retrospective design and the heterogeneity between the studies limit the strength of this type of study, and the sensitivity analysis often alters the results obtained in a first evaluation [90]. Consequently, we cannot state that these biomarkers are better than others available to assess the prognosis of these patients [68,69,70], which merits further studies on this topic.

### Limitations

Our study has several limitations. First, high statistical heterogeneity was found due to clinical and methodological differences between studies. However, the heterogeneity decreased when sensitivity analysis was performed. Second, most of the studies were carried out in Turkey, which means that the results could probably not be applied to all countries. Thus, it would be beneficial to analyze the prognostic value of these biomarkers in more countries. Finally, due to the lack of information in the studies, it was not possible to reach a consensus to determine an optimal cut-off point of FAR and BAR for the risk of severity or mortality because the oscillation of values was highly variable. Hence, it would be important to address it in future studies in various populations with different sociodemographic characteristics and lifestyles.

## 5. Conclusions

COVID-19 patients with high FAR and BAR values were at high risk of mortality. However, only high FAR values were associated with a higher risk of severe disease. Further primary studies are needed to define the optimal cut-off point for these markers and reach a consensus on their prognostic value.

## Figures and Tables

**Table 1:** Characteristics of the included studies that evaluated severity.

Author	Year	Country	*Participants* *(Male)*	*Median/Mean Age (IQR/SD)*	Marker Analyzed	Marker Mean (SD) in Severe Patients	Marker Mean (SD) in Non-Severe Patients	Odds Ratio [95% CI]	Cut-Off	Area under the Curve	Sensivity (%)	Specificity (%)
*Gemcioglu et al.*	2021	Turkey	301 (161)	49 (26.5)	FAR	NR	NR	1 [0.53–1.88]	0.102	0.766	65.31%	77.91%
					BAR	NR	NR	2.18 [1.15–4.15]	4.78	0.795	63.37%	84.89%
*Kuluöztürk et al.*	2021	Turkey	400 (235)	55.51 (18.88)	FAR	NR	NR	2.76 [1.31–5.79]	0.144	0.654	72%	53%
*Bi et al.*	2020	China	113 (64)	46 (37–55)	FAR	NR	NR	5.81 [1.28–26.34]	0.088	0.73	NR	NR
*Torun et al.*	2021	Turkey	188 (95)	62.3 (12.7)	FAR	0.14 (0.17)	0.12 (0.18)	1.18 [0.70–1.99]	0.113	0.737	69.6%	65.8%
*Yang et al.*	2021	China	495 (235)	55 (40–67)	FAR	0.134 (0.04)	0.104 (0.034)	4.41 [3.13–6.22]	0.12	0.838	80.8%	64%
*Lawson et al.*	2022	Nigeria	600 (374)	42.2 (6.71)	FAR	NR	NR	1.19 [0.73–1.94]	NR	NR	NR	NR
*Huang et al.*	2021	China	1370 (328)	55 (40–66)	BAR	NR	NR	1.32 [1.18–1.47]	3.788	0.821	68%	78.6%
*Nie et al.*	2020	China	97 (34)	39 (30–60)	BAR	1 (0.44)	1.02 (0.22)	0.79 [0.49–1.28]	NR	NR	NR	NR
*Alirezaei et al.*	2022	Iran	433 (263)	60.38 (18.26)	BAR	4.15 (2.81)	4.32 (2.74)	0.90 [0.59–1.36]	3.954	0.475	47.5%	40.6%
*Yazıcı et al.*	2022	Turkey	252 (107)	77 (70–83)	FAR	0.185 (0.04)	0.131 (0.04)	9.43 [4.83–18.43]	0.15	0.789	84.2%	69.6%
*Çalışkan et al.*	2022	Turkey	548 (286)	64 (21)	FAR	13.65 (7.88)	11.7 (4.39)	1.95 [1.34–2.86]	0.147	0.629	83.23%	45.31%

NR: Not reported; 95% CI: 95% Confidence interval; IQR: Interquartile range; SD: Standard deviation.

**Table 2:** Characteristics of the included studies that evaluated the mortality.

Author	Year	Country	*Participants* *(Male)*	*Median/Mean Age (IQR/SD)*	Marker Analyzed	Marker Mean (SD) in Non-Survivors	Marker Mean (SD) in Survivors	Odds Ratio [95% CI]	Cut-Off	Area under the Curve	Sensivity (%)	Specificity (%)
*Kuluöztürk et al.*	2021	Turkey	400 (235)	55.51 (18.88)	FAR	NR	NR	3.06 [1.33–7.07]	0.144	0.654	72%	53%
*Afşin et al.*	2021	Turkey	386 (209)	71.28 (12.9)	FAR	NR	NR	1 [1–1.01]	NR	NR	NR	NR
*Atlas et al.*	2021	Turkey	102 (74)	69.1 (14.3)	FAR	0.202 (0.037)	0.13 (0.014)	50.79 [18.77–137.44]	0.15	0.989	NR	NR
*Küçükceran et al.* [38]	2021	Turkey	717 (371)	64 (50–74)	FAR	NR	NR	4.44 [2.91–6.76]	0.1123	0.703	71.4%	64%
*Küçükceran et al.* [46]	2021	Turkey	602 (312)	63 (49–73)	BAR	NR	NR	10.45 [5.56–19.63]	3.9	0.809	87.5%	59.9%
*Çekiç et al.*	2021	Turkey	590 (358)	65.63 (14.9)	FAR	0.14 (0.17)	0.12 (0.18)	1.63 [1.20–2.22]	0.13	0.808	74.9%	74.6%
*Yang et al.*	2021	China	495 (235)	55 (40–67)	FAR	NR	NR	3.29 [1.55–7.01]	0.12	0.838	80.8%	64%
*Acehan et al.*	2021	Turkey	613 (358)	59.04 (19.5)	FAR	NR	NR	1.01 [0.96–1.06]	0.111	0.668	62.3%	57.5%
*Ata et al.*	2021	Turkey	358 (148)	66 (50.5–77)	BAR	NR	NR	2.69 [2.02–3.59]	3.4	0.823	74.5%	75.6%
*Singh et al.*	2022	India	131 (98)	54 (14)	BAR	NR	NR	3.75 [1.66–8.47]	6.23	0.695	79%	54%
*Mihić et al.*	2022	Croatia	138 (NR)	68 (38–88)	FAR	NR	NR	1.30 [0.61–2.76]	NR	NR	NR	NR
*Alirezaei et al.*	2022	Iran	433 (263)	60.38 (18.26)	BAR	9.27 (7.03)	3.8 (2.07)	11.31 [7.46–17.14]	4.944	0.758	75.8%	70.8%
*Yazıcı et al.*	2022	Turkey	252 (107)	77 (70–83)	FAR	0.173 (0.05)	0.128 (0.03)	6.69 [3.96–11.31]	0.144	0.731	75%	69%
*Olgun et al.*	2022	Turkey	117 (74)	62.65 (15.89)	BAR	12.76 (35.45)	4.76 (35.59)	1.49 [0.58–3.82]	NR	NR	NR	NR

## Data Availability

Not applicable.

## Supplementary Materials

The following supporting information can be downloaded at: https://www.mdpi.com/article/10.3390/tropicalmed7080150/s1, Table S1: PRISMA Checklist; Table S2: Search Strategy; Table S3: Newcastle—Ottawa Quality assessment scale for included studies; Figure S1: Subgroup analysis according to countries of the association between FAR and severity of COVID-19 patients; Figure S2: Sensitivity analysis of the association between FAR and severity of COVID-19 patients; Figure S3: Subgroup analysis according to countries of the association between FAR and mortality of COVID-19 patients; Figure S4: Sensitivity analysis of the association between FAR and mortality of COVID-19 patients
