# Supplementary material for: Fibrinogen-to-Albumin Ratio and Blood Urea Nitrogen-to-Albumin Ratio in COVID-19 Patients: A Systematic Review and Meta-Analysis
Source: Trop Med Infect Dis. 2022 Jul 27;7(8):150. doi: 10.3390/tropicalmed7080150 (PMC9414552; doi:10.3390/tropicalmed7080150)

SUPPLEMENTARY INFORMATION

Table S1. PRISMA Checklist.

Section and Topic	Item #	Checklist item	Location where item is reported
**TITLE**			
Title	1	Identify the report as a systematic review.	Title Page
**ABSTRACT**			
Abstract	2	See the PRISMA 2020 for Abstracts checklist.	Title Page
**INTRODUCTION**			
Rationale	3	Describe the rationale for the review in the context of existing knowledge.	Fifth paragraphof the introduction section
Objectives	4	Provide an explicit statement of the objective(s) or question(s) the review addresses.	Fifth paragraph of the introduction section
**METHODS**			
Eligibility criteria	5	Specify the inclusion and exclusion criteria for the review and how studies were grouped for the syntheses.	Subheading 2.2
Information sources	6	Specify all databases, registers, websites, organisations, reference lists and other sources searched or consulted to identify studies. Specify the date when each source was last searched or consulted.	Subheading 2.1
Search strategy	7	Present the full search strategies for all databases, registers and websites, including any filters and limits used.	Subheading 2.1 and Supplementary Table S2
Selection process	8	Specify the methods used to decide whether a study met the inclusion criteria of the review, including how many reviewers screened each record and each report retrieved, whether they worked independently, and if applicable, details of automation tools used in the process.	Subheading 2.3
Data collection process	9	Specify the methods used to collect data from reports, including how many reviewers collected data from each report, whether they worked independently, any processes for obtaining or confirming data from study investigators, and if applicable, details of automation tools used in the process.	Subheading 2.3
Data items	10a	List and define all outcomes for which data were sought. Specify whether all results that were compatible with each outcome domain in each study were sought (e.g. for all measures, time points, analyses), and if not, the methods used to decide which results to collect.	Subheading 2.6

Section and Topic	Item #	Checklist item	Location where item is reported
	10b	List and define all other variables for which data were sought (e.g. participant and intervention characteristics, funding sources). Describe any assumptions made about any missing or unclear information.	Subheading 2.6
Study risk of bias assessment	11	Specify the methods used to assess risk of bias in the included studies, including details of the tool(s) used, how many reviewers assessed each study and whether they worked independently, and if applicable, details of automation tools used in the process.	Subheadings 2.4 and 2.6
Effect measures	12	Specify for each outcome the effect measure(s) (e.g. risk ratio, mean difference) used in the synthesis or presentation of results.	Subheading 2.6
Synthesis methods	13a	Describe the processes used to decide which studies were eligible for each synthesis (e.g. tabulating the study intervention characteristics and comparing against the planned groups for each synthesis (item #5)).	Subheading 2.6
	13b	Describe any methods required to prepare the data for presentation or synthesis, such as handling of missing summary statistics, or data conversions.	Subheading 2.6
	13c	Describe any methods used to tabulate or visually display results of individual studies and syntheses.	Subheading 2.6
	13d	Describe any methods used to synthesize results and provide a rationale for the choice(s). If meta-analysis was performed, describe the model(s), method(s) to identify the presence and extent of statistical heterogeneity, and software package(s) used.	Subheading 2.6
	13e	Describe any methods used to explore possible causes of heterogeneity among study results (e.g. subgroup analysis, meta-regression).	Subheading 2.6
	13f	Describe any sensitivity analyses conducted to assess robustness of the synthesized results.	Subheading 2.6
Reporting bias assessment	14	Describe any methods used to assess risk of bias due to missing results in a synthesis (arising from reporting biases).	Subheading 2.4
Certainty assessment	15	Describe any methods used to assess certainty (or confidence) in the body of evidence for an outcome.	Subheading 2.6
**RESULTS**			
Study selection	16a	Describe the results of the search and selection process, from the number of records identified in the search to the number of studies included in the review, ideally using a flow diagram.	Subheading 3.1
	16b	Cite studies that might appear to meet the inclusion criteria, but which were excluded, and explain why they were excluded.	Subheading 3.1
Study characteristics	17	Cite each included study and present its characteristics.	Subheading 3.2

Section and Topic	Item #	Checklist item	Location where item is reported
Risk of bias in studies	18	Present assessments of risk of bias for each included study.	Subheading 3.2
Results of individual studies	19	For all outcomes, present, for each study: (a) summary statistics for each group (where appropriate) and (b) an effect estimate and its precision (e.g. confidence/credible interval), ideally using structured tables or plots.	Subheadings 3.3 , 3.4 , 3.5 and 3.6
Results of syntheses	20a	For each synthesis, briefly summarise the characteristics and risk of bias among contributing studies.	Subheadings 3.3 , 3.4 , 3.5 and 3.6
	20b	Present results of all statistical syntheses conducted. If meta-analysis was done, present for each the summary estimate and its precision (e.g. confidence/credible interval) and measures of statistical heterogeneity. If comparing groups, describe the direction of the effect.	Subheadings 3.3 , 3.4 , 3.5 and 3.6
	20c	Present results of all investigations of possible causes of heterogeneity among study results.	Subheadings 3.3 , 3.4 , 3.5 and 3.6
	20d	Present results of all sensitivity analyses conducted to assess the robustness of the synthesized results.	Subheadings 3.3 , 3.4 , 3.5 and 3.6
Reporting biases	21	Present assessments of risk of bias due to missing results (arising from reporting biases) for each synthesis assessed.	Subheadings 3.2 and 3.7
Certainty of evidence	22	Present assessments of certainty (or confidence) in the body of evidence for each outcome assessed.	Subheadings 3.3 , 3.4 , 3.5 and 3.6
**DISCUSSION**			
Discussion	23a	Provide a general interpretation of the results in the context of other evidence.	Line 1-3 of the Discussion section
	23b	Discuss any limitations of the evidence included in the review.	Subheading 4.1
	23c	Discuss any limitations of the review processes used.	Subheading 4.1
	23d	Discuss implications of the results for practice, policy, and future research.	Subheading 4.1
**OTHER INFORMATION**			
Registration and protocol	24a	Provide registration information for the review, including register name and registration number, or state that the review was not registered.	First paragraph of section 2
	24b	Indicate where the review protocol can be accessed, or state that a protocol was not prepared.	First paragraph of section 2
	24c	Describe and explain any amendments to information provided at registration or in the protocol.	First paragraph of section 2
Support	25	Describe sources of financial or non-financial support for the review, and the role of the funders or sponsors in the review.	Page 13 in Statements and Declarations
Competing interests	26	Declare any competing interests of review authors.	Page 13 in Statements and Declarations

Section and Topic	Item #	Checklist item	Location where item is reported
Availability of data, code and other materials	27	Report which of the following are publicly available and where they can be found: template data collection forms; data extracted from included studies; data used for all analyses; analytic code; any other materials used in the review.	Page 13 in Statements and Declarations

Table S2. Search strategy.

**Source**	**PubMed**
**Search**	**Formula**
**#1**	Fibrinogen [MH] OR "fibrinogen" [TIAB] OR (("factor i" [TIAB] OR "factor 1" [TIAB]) AND ("clott*" [TIAB] OR "coagulation" [TIAB])) OR "fibclot" [TIAB] OR "clottagen" [TIAB] OR "fibryga" [TIAB]
**#2**	Albumin [MH] OR "albumin*" [TIAB] OR "albumen*" [TIAB]
**#3**	((Fibrinogen [MH] OR "fibrinogen" [TIAB] OR (("factor i" [TIAB] OR "factor 1" [TIAB]) AND ("clott*" [TIAB] OR "coagulation" [TIAB])) OR "fibclot" [TIAB] OR "clottagen" [TIAB] OR "fibryga" [TIAB]) AND (Albumin [MH] OR "albumin*" [TIAB] OR "albumen*" [TIAB])) AND ("ratio" OR "index")
**#4**	(("blood" [TIAB] OR "plasma" [TIAB] OR "serum" [TIAB]) AND ("urea" [TIAB] AND "nitrogen" [TIAB])) OR "BUN" [TIAB]
**#5**	((((("blood" [TIAB] OR "plasma" [TIAB] OR "serum" [TIAB]) AND ("urea" [TIAB] AND "nitrogen" [TIAB])) OR "BUN" [TIAB]) AND (Albumin [MH] OR "albumin*" [TIAB] OR "albumen*" [TIAB])) AND ("ratio" OR "index"))
**#6**	COVID-19 [MH] OR "covid*" [TIAB] OR (("covid*" [TIAB] OR "coronavirus" [TIAB] OR "ncov*" [TIAB] OR "cov*" [TIAB] OR "sars*" [TIAB]) AND ("infection*" [TIAB] OR "disease*" [TIAB] OR "pandemic*" [TIAB] OR "epidemic" [TIAB] OR "severe acute respiratory syndrome" [TIAB] OR "pneumonia*" [TIAB]))
**#7**	#1 AND #2
**#8**	#3 OR #7
**#9**	#2 AND #4

**#10**	#5 OR #9
**#11**	#8 OR #10
**#12**	#6 AND #11
**Source**	**Scopus**
**Search**	**Formula**
**#1**	TITLE-ABS-KEY (“fibrinogen” OR ((“factor i” OR “factor 1”) W/3 (“clott*” OR “coagulation”)) OR “fibclot” OR “clottagen” OR “fibryga”)
**#2**	TITLE-ABS-KEY (“albumin*” OR “albumen*”)
**#3**	TITLE-ABS-KEY (((“fibrinogen” OR ((“factor i” OR “factor 1”) W/3 (“clott*” OR “coagulation”)) OR “fibclot” OR “clottagen” OR “fibryga”) W/3 (“albumin*” OR “albumen*”)) W/3 (“ratio” OR “index”))
**#4**	TITLE-ABS-KEY (((“blood” OR “plasma” OR “serum”) W/3 (“urea” W/3 “nitrogen”)) OR “BUN”)
**#5**	TITLE-ABS-KEY ((((((“blood” OR “plasma” OR “serum”) W/3 (“urea” W/3 “nitrogen”)) OR “BUN”) W/3 (“albumin*” OR “albumen*”)) W/3 (“ratio” OR “index”)))
**#6**	TITLE-ABS-KEY (“covid*” OR ((“covid*” OR “coronavirus” OR “ncov*” OR “cov*” OR “sars*”) W/3 (“infection*” OR “disease*” OR “pandemic*” OR “epidemic” OR “severe acute respiratory syndrome” OR “pneumonia*”)))
**#7**	#1 AND #2
**#8**	#3 OR #7
**#9**	#2 AND #4
**#10**	#5 OR #9
**#11**	#8 OR #10
**#12**	#6 AND #11
**Source**	**Web of Science**
**Search**	**Formula**

**#1**	TI=(“fibrinogen” OR ((“factor i” OR “factor 1”) NEAR/3 (“clott*” OR “coagulation”)) OR “fibclot” OR “clottagen” OR “fibryga”) OR AB=(“fibrinogen” OR ((“factor i” OR “factor 1”) NEAR/3 (“clott*” OR “coagulation”)) OR “fibclot” OR “clottagen” OR “fibryga”) OR AK=(“fibrinogen” OR ((“factor i” OR “factor 1”) NEAR/3 (“clott*” OR “coagulation”)) OR “fibclot” OR “clottagen” OR “fibryga”) OR KP=(“fibrinogen” OR ((“factor i” OR “factor 1”) NEAR/3 (“clott*” OR “coagulation”)) OR “fibclot” OR “clottagen” OR “fibryga”) OR TS=(“fibrinogen” OR ((“factor i” OR “factor 1”) NEAR/3 (“clott*” OR “coagulation”)) OR “fibclot” OR “clottagen” OR “fibryga”)
**#2**	TI=(“albumin*” OR “albumen*”) OR AB=(“albumin*” OR “albumen*”) OR AK=(“albumin*” OR “albumen*”) OR KP=(“albumin*” OR “albumen*”) OR TS=(“albumin*” OR “albumen*”)
**#3**	TI=(((“fibrinogen” OR ((“factor i” OR “factor 1”) NEAR/3 (“clott*” OR “coagulation”)) OR “fibclot” OR “clottagen” OR “fibryga”) NEAR/3 (“albumin*” OR “albumen*”)) NEAR/3 (“ratio” OR “index”)) OR AB=(((“fibrinogen” OR ((“factor i” OR “factor 1”) NEAR/3 (“clott*” OR “coagulation”)) OR “fibclot” OR “clottagen” OR “fibryga”) NEAR/3 (“albumin*” OR “albumen*”)) NEAR/3 (“ratio” OR “index”)) OR AK=(((“fibrinogen” OR ((“factor i” OR “factor 1”) NEAR/3 (“clott*” OR “coagulation”)) OR “fibclot” OR “clottagen” OR “fibryga”) NEAR/3 (“albumin*” OR “albumen*”)) NEAR/3 (“ratio” OR “index”)) OR KP=(((“fibrinogen” OR ((“factor i” OR “factor 1”) NEAR/3 (“clott*” OR “coagulation”)) OR “fibclot” OR “clottagen” OR “fibryga”) NEAR/3 (“albumin*” OR “albumen*”)) NEAR/3 (“ratio” OR “index”)) OR TS=(((“fibrinogen” OR ((“factor i” OR “factor 1”) NEAR/3 (“clott*” OR “coagulation”)) OR “fibclot” OR “clottagen” OR “fibryga”) NEAR/3 (“albumin*” OR “albumen*”)) NEAR/3 (“ratio” OR “index”))
**#4**	TI=(((“blood” OR “plasma” OR “serum”) NEAR/3 (“urea” NEAR/3 “nitrogen”)) OR “BUN”) OR AB=(((“blood” OR “plasma” OR “serum”) NEAR/3 (“urea” NEAR/3 “nitrogen”)) OR “BUN”) OR AK=(((“blood” OR

	“plasma” OR “serum”) NEAR/3 (“urea” NEAR/3 “nitrogen”)) OR “BUN”) OR KP=(((“blood” OR “plasma” OR “serum”) NEAR/3 (“urea” NEAR/3 “nitrogen”)) OR “BUN”) OR TS=(((“blood” OR “plasma” OR “serum”) NEAR/3 (“urea” NEAR/3 “nitrogen”)) OR “BUN”)
**#5**	TI=((((((“blood” OR “plasma” OR “serum”) NEAR/3 (“urea” NEAR/3 “nitrogen”)) OR “BUN”) NEAR/3 (“albumin*” OR “albumen*”)) NEAR/3 (“ratio” OR “index”))) OR AB=((((((“blood” OR “plasma” OR “serum”) NEAR/3 (“urea” NEAR/3 “nitrogen”)) OR “BUN”) NEAR/3 (“albumin*” OR “albumen*”)) NEAR/3 (“ratio” OR “index”))) OR AK=((((((“blood” OR “plasma” OR “serum”) NEAR/3 (“urea” NEAR/3 “nitrogen”)) OR “BUN”) NEAR/3 (“albumin*” OR “albumen*”)) NEAR/3 (“ratio” OR “index”))) OR KP=((((((“blood” OR “plasma” OR “serum”) NEAR/3 (“urea” NEAR/3 “nitrogen”)) OR “BUN”) NEAR/3 (“albumin*” OR “albumen*”)) NEAR/3 (“ratio” OR “index”))) OR TS=((((((“blood” OR “plasma” OR “serum”) NEAR/3 (“urea” NEAR/3 “nitrogen”)) OR “BUN”) NEAR/3 (“albumin*” OR “albumen*”)) NEAR/3 (“ratio” OR “index”)))
**#6**	TI=(“covid*” OR ((“covid*” OR “coronavirus” OR “ncov*” OR “cov*” OR “sars*”) NEAR/3 (“infection*” OR “disease*” OR “pandemic*” OR “epidemic” OR “severe acute respiratory syndrome” OR “pneumonia*”))) OR AB=(“covid*” OR ((“covid*” OR “coronavirus” OR “ncov*” OR “cov*” OR “sars*”) NEAR/3 (“infection*” OR “disease*” OR “pandemic*” OR “epidemic” OR “severe acute respiratory syndrome” OR “pneumonia*”))) OR AK=(“covid*” OR ((“covid*” OR “coronavirus” OR “ncov*” OR “cov*” OR “sars*”) NEAR/3 (“infection*” OR “disease*” OR “pandemic*” OR “epidemic” OR “severe acute respiratory syndrome” OR “pneumonia*”))) OR KP=(“covid*” OR ((“covid*” OR “coronavirus” OR “ncov*” OR “cov*” OR “sars*”) NEAR/3 (“infection*” OR “disease*” OR “pandemic*” OR “epidemic” OR “severe acute respiratory syndrome” OR “pneumonia*”))) OR TS=(“covid*” OR ((“covid*” OR “coronavirus” OR “ncov*” OR “cov*” OR “sars*”) NEAR/3 (“infection*” OR “disease*” OR “pandemic*” OR “epidemic” OR “severe acute respiratory syndrome” OR “pneumonia*”)))

**#7**	#1 AND #2
**#8**	#3 OR #7
**#9**	#2 AND #4
**#10**	#5 OR #9
**#11**	#8 OR #10
**#12**	#6 AND #11
**Source**	**Embase**
**Search**	**Formula**
**#1**	((“fibrinogen” OR ((“factor i” OR “factor 1”) NEAR/3 (“clott*” OR “coagulation”)) OR “fibclot” OR “clottagen” OR “fibryga”):ti) OR ((“fibrinogen” OR ((“factor i” OR “factor 1”) NEAR/3 (“clott*” OR “coagulation”)) OR “fibclot” OR “clottagen” OR “fibryga”):ab) OR ((“fibrinogen” OR ((“factor i” OR “factor 1”) NEAR/3 (“clott*” OR “coagulation”)) OR “fibclot” OR “clottagen” OR “fibryga”):kw)
**#2**	((“albumin*” OR “albumen*”):ti) OR ((“albumin*” OR “albumen*”):ab) OR ((“albumin*” OR “albumen*”):kw)
**#3**	
**#4**	(():ti) OR (():ab) OR (():kw)
**#5**	(():ti) OR (():ab) OR (():kw)
**#6**	(():ti) OR (():ab) OR (():kw)
**#7**	#1 AND #2
**#8**	#3 OR #7
**#9**	#2 AND #4
**#10**	#5 OR #9
**#11**	#8 OR #10
**#12**	#6 AND #11
**Source**	**OVID**

Search	Formula
**#1**	(fibrinogen OR ((factor i OR factor 1) adj3 (clott* OR coagulation)) OR fibclot OR clottagen OR fibryga).ti. OR (fibrinogen OR ((factor i OR factor 1) adj3 (clott* OR coagulation)) OR fibclot OR clottagen OR fibryga).ab. OR (fibrinogen OR ((factor i OR factor 1) adj3 (clott* OR coagulation)) OR fibclot OR clottagen OR fibryga).kw.
**#2**	(albumin* OR albumen*).ti. OR (albumin* OR albumen*).ab. OR (albumin* OR albumen*).kw.
**#3**	(((fibrinogen OR ((factor i OR factor 1) adj3 (clott* OR coagulation)) OR fibclot OR clottagen OR fibryga) adj3 (albumin* OR albumen*)) adj3 (ratio OR index)).ti. OR (((fibrinogen OR ((factor i OR factor 1) adj3 (clott* OR coagulation)) OR fibclot OR clottagen OR fibryga) adj3 (albumin* OR albumen*)) adj3 (ratio OR index)).ab. OR (((fibrinogen OR ((factor i OR factor 1) adj3 (clott* OR coagulation)) OR fibclot OR clottagen OR fibryga) adj3 (albumin* OR albumen*)) adj3 (ratio OR index)).kw.
**#4**	(((blood OR plasma OR serum) adj3 (urea adj3 nitrogen)) OR BUN).ti. OR (((blood OR plasma OR serum) adj3 (urea adj3 nitrogen)) OR BUN).ab. OR (((blood OR plasma OR serum) adj3 (urea adj3 nitrogen)) OR BUN).kw.
**#5**	((((((blood OR plasma OR serum) adj3 (urea adj3 nitrogen)) OR BUN) adj3 (albumin* OR albumen*)) adj3 (ratio OR index))).ti. OR ((((((blood OR plasma OR serum) adj3 (urea adj3 nitrogen)) OR BUN) adj3 (albumin* OR albumen*)) adj3 (ratio OR index))).ab. OR ((((((blood OR plasma OR serum) adj3 (urea adj3 nitrogen)) OR BUN) adj3 (albumin* OR albumen*)) adj3 (ratio OR index))).kw.
**#6**	(covid* OR ((covid* OR coronavirus OR ncov* OR cov* OR sars*) adj3 (infection* OR disease* OR pandemic* OR epidemic OR severe acute respiratory syndrome OR pneumonia*))).ti. OR (covid* OR ((covid* OR coronavirus OR ncov* OR cov* OR sars*) adj3 (infection* OR disease* OR pandemic* OR epidemic OR severe acute respiratory syndrome OR pneumonia*))).ab. OR (covid* OR ((covid* OR coronavirus OR ncov* OR

	cov* OR sars*) adj3 (infection* OR disease* OR pandemic* OR epidemic OR severe acute respiratory syndrome OR pneumonia*))).kw.
**#7**	#1 AND #2
**#8**	#3 OR #7
**#9**	#2 AND #4
**#10**	#5 OR #9
**#11**	#8 OR #10
**#12**	#6 AND #11

Table S3. Newcastle - Ottawa Quality assessment scale for included studies.

NEWCASTLE - OTTAWA QUALITY ASSESSMENT SCALE FOR COHORT STUDIES										
STUDY	**SELECTION**				**COMPARABILITY**	**OUTCOME**				
	**Representativeness of the exposed cohort**	**Selection of the non-exposed cohort**	**Ascertainment of exposure**	**Demonstration that outcome of interest was not present at start of study**	**Comparability of Cohorts on the Basis of the Design or Analysis Maximum : ☆☆**	**Assessment of outcome**	**Was follow-up long enough for outcomes to occur**	**Adequacy of follow up of cohorts**	**SCORE**	**Evidence quality**
Gemcioglu et.al	☆		☆			☆	☆	☆	*5*	High Risk of Bias
Afşin et.al	☆		☆			☆	☆	☆	*5*	High Risk of Bias
Kuluöztürk et.al	☆	☆	☆	☆	☆	☆	☆	☆	*8*	Low Risk of bias
Bi et.al	☆	☆	☆	☆		☆	☆	☆	*7*	Low Risk of bias
Torun et.al		☆	☆			☆	☆	☆	*5*	High Risk of Bias
Yang et.al	☆	☆	☆	☆	☆☆	☆	☆	☆	*9*	Low Risk of bias

Lawson et.al		☆	☆			☆	☆	☆	*5*	High Risk of Bias
Huang et.al	☆	☆	☆	☆		☆	☆	☆	*7*	Low Risk of bias
Nie et.al	☆	☆	☆			☆	☆	☆	*6*	Low Risk of bias
Atlas et.al		☆	☆			☆	☆	☆	*5*	High Risk of Bias
Küçükceran (38) et.al		☆	☆			☆	☆	☆	*5*	High Risk of Bias
Küçükceran (46) et.al		☆	☆			☆	☆	☆	*5*	High Risk of Bias
Çekiç et.al	☆	☆	☆	☆	☆	☆	☆	☆	*8*	Low Risk of bias
Acehan et.al		☆	☆			☆	☆	☆	*5*	High Risk of Bias
Ata et.al	☆	☆	☆			☆	☆	☆	*6*	Low Risk of bias
Singh et.al	☆	☆	☆			☆	☆	☆	*6*	Low Risk of bias
Mihić et.al	☆	☆	☆	☆	☆	☆	☆	☆	*8*	Low Risk of bias

Alirezaei et.al	☆	☆	☆	☆	☆☆	☆	☆	☆	*9*	Low Risk of bias
Yazıcı et.al	☆		☆			☆	☆	☆	*5*	High Risk of Bias
Çalışkan et.al		☆	☆			☆	☆	☆	*5*	High Risk of Bias
Olgun et.al		☆	☆			☆	☆	☆	*5*	High Risk of Bias
Çelikkol A et.al		☆	☆			☆	☆	☆	*5*	High Risk of Bias
Çalışkan Z et.al	☆		☆			☆	☆	☆	*5*	High Risk of Bias
Ergenç Z et.al		☆	☆			☆	☆	☆	*5*	High Risk of Bias
Yazıcı et.al		☆	☆			☆	☆	☆	*5*	High Risk of Bias
Katkat F et.al		☆	☆			☆	☆	☆	*5*	High Risk of Bias
Prasad S et.al		☆	☆			☆	☆	☆	*5*	High Risk of Bias
Hocanlı I et.al	☆		☆			☆	☆	☆	*5*	High Risk of Bias

Arslan K et.al		☆	☆			☆	☆	☆	*5*	High Risk of Bias
Gozdas H et.al		☆	☆			☆	☆	☆	*5*	High Risk of Bias

Figure S1. Subgroup analysis according to countries of the association between FAR and severity of COVID-19 patients.

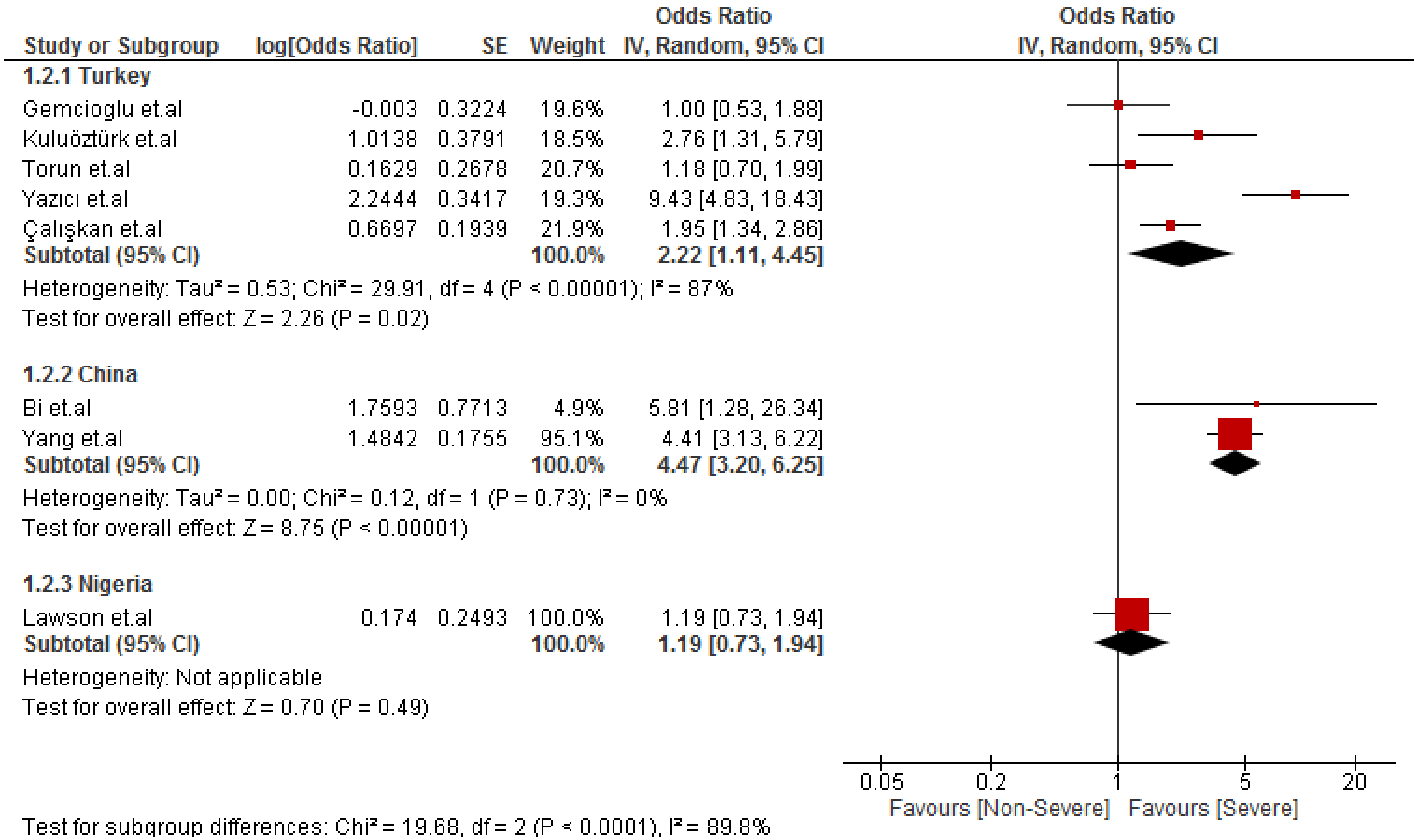

Figure S2. Sensitivity analysis of the association between FAR and severity of COVID-19 patients.

Study or Subgroup	log[Odds Ratio]	SE	Weight	Odds Ratio IV, Random, 95% CI
**1.1.1 Low Risk of bias**				
Bi et.al	1.7593	0.7713	4.1%	5.81 [1.28, 26.34]
Kuluöztürk et.al	1.0138	0.3791	16.9%	2.76 [1.31, 5.79]
Yang et.al	1.4842	0.1755	79.0%	4.41 [3.13, 6.22]
**Subtotal (95% CI)**			**100.0%**	**4.12 [3.03, 5.59]**

Heterogeneity: Tau² = 0.00; Chi² = 1.47, df = 2 (P = 0.48); I² = 0%
Test for overall effect: Z = 9.08 (P < 0.00001)

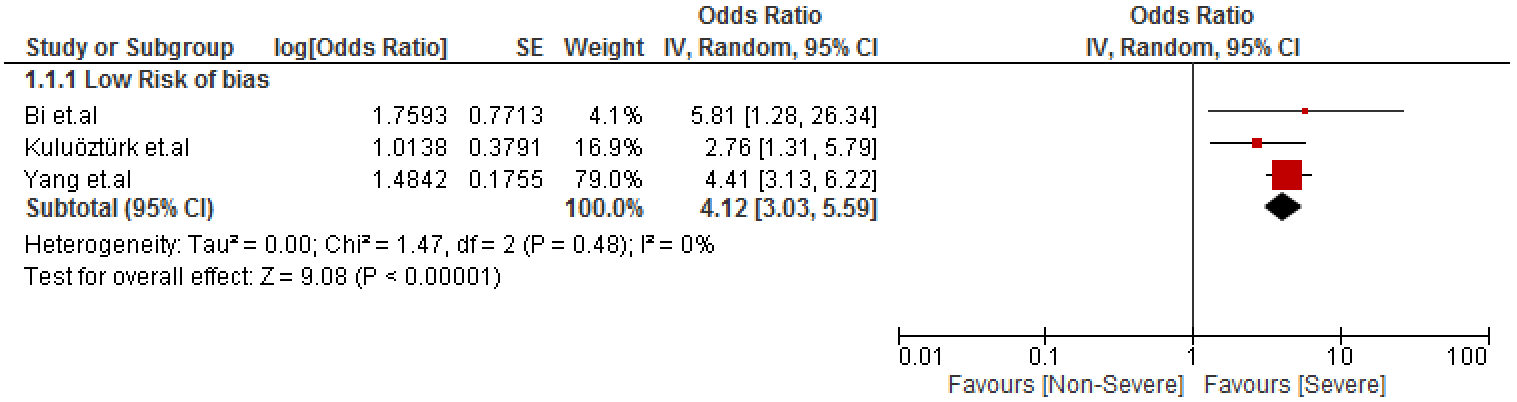

Figure S3. Subgroup analysis according to countries of the association between FAR and mortality of COVID-19 patients.

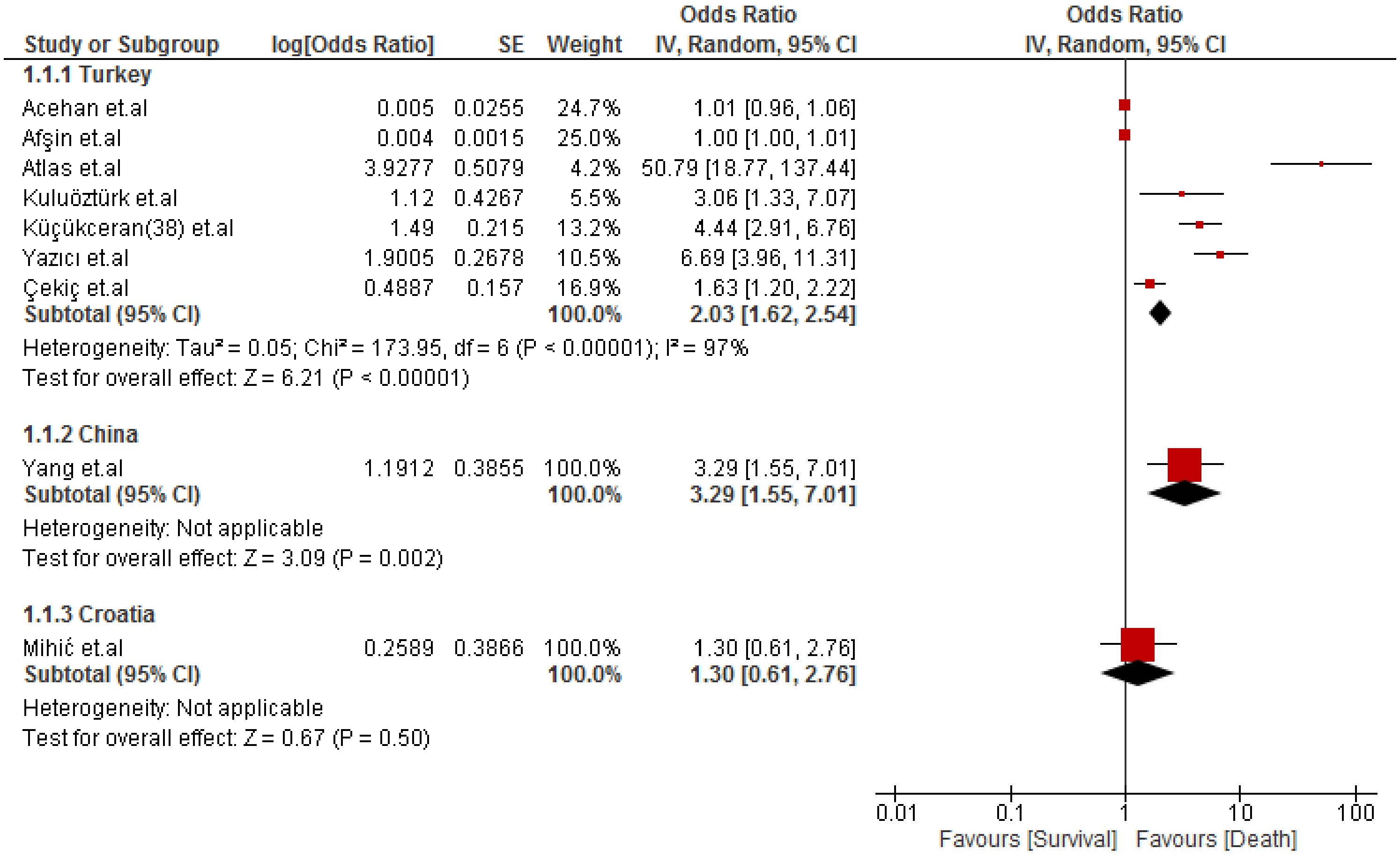

Figure S4. Sensitivity analysis of the association between FAR and mortality of COVID-19 patients.

Study or Subgroup	log[Odds Ratio]	SE	Weight	Odds Ratio IV, Random, 95% CI
**1.1.1 Low Risk of bias**				
Kuluöztürk et.al	1.12	0.4267	16.8%	3.06 [1.33, 7.07]
Mihić et.al	0.2589	0.3866	19.3%	1.30 [0.61, 2.76]
Yang et.al	1.1912	0.3855	19.4%	3.29 [1.55, 7.01]
Çekiç et.al	0.4887	0.157	44.5%	1.63 [1.20, 2.22]
**Subtotal (95% CI)**			**100.0%**	**1.99 [1.33, 2.98]**

Heterogeneity: Tau² = 0.07; Chi² = 5.12, df = 3 (P = 0.16); I² = 41%

Test for overall effect: Z = 3.33 (P = 0.0009)

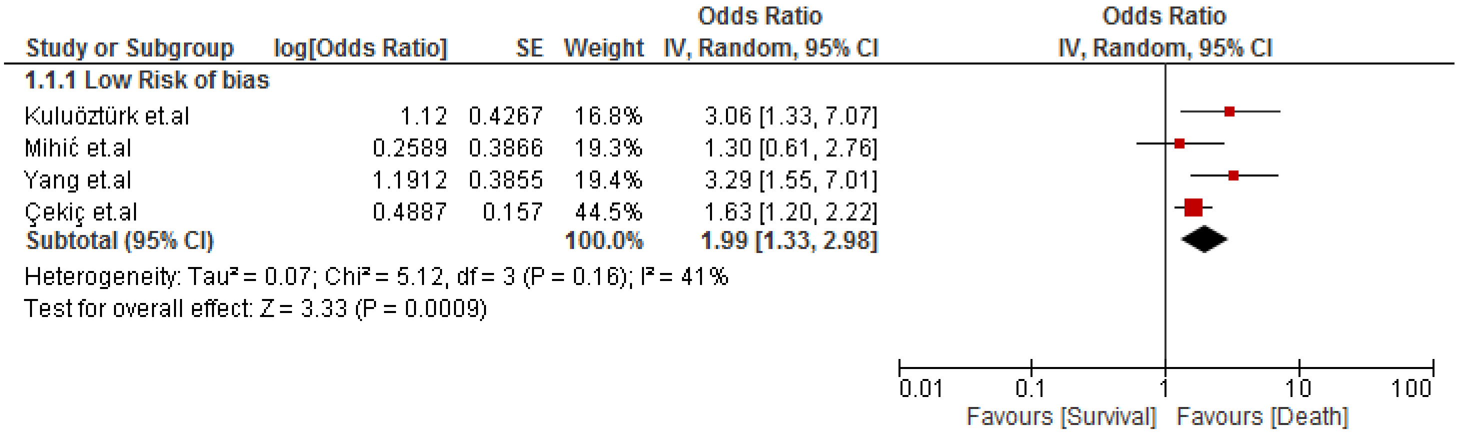

Supplement: Supplementary file 1 [file tropicalmed-07-00150-s001.zip › tropicalmed-1790048-supplementary.pdf]
